# Different strategies for spatial updating in yaw and pitch path integration

**DOI:** 10.3389/fnbeh.2013.00005

**Published:** 2013-02-13

**Authors:** Caspar M. Goeke, Peter König, Klaus Gramann

**Affiliations:** ^1^Department of Neurobiopsychology, Institute of Cognitive Science, University of OsnabrückOsnabrück, Germany; ^2^Department of Neurophysiology and Pathophysiology, University Medical Center Hamburg-EppendorfHamburg, Germany; ^3^Biological Psychology und Neuroergonomics, Technical University BerlinBerlin, Germany; ^4^Center for Advanced Neurological Engineering, Institute for Neural Computation, University of CaliforniaSan Diego, CA, USA

**Keywords:** spatial navigation, reference frames, path integration, gender differences, navigational strategies

## Abstract

Research in spatial navigation revealed the existence of discrete strategies defined by the use of distinct reference frames during virtual path integration. The present study investigated the distribution of these navigation strategies as a function of gender, video gaming experience, and self-estimates of spatial navigation abilities in a population of 300 subjects. Participants watched videos of virtual passages through a star-field with one turn in either the horizontal (yaw) or the vertical (pitch) axis. At the end of a passage they selected one out of four homing arrows to indicate the initial starting location. To solve the task, participants could employ two discrete strategies, navigating within either an egocentric or an allocentric reference frame. The majority of valid subjects (232/260) consistently used the same strategy in more than 75% of all trials. With that approach 33.1% of all participants were classified as Turners (using an egocentric reference frame on both axes) and 46.5% as Non-turners (using an allocentric reference frame on both axes). 9.2% of all participants consistently used an egocentric reference frame in the yaw plane but an allocentric reference frame in the pitch plane (Switcher). Investigating the influence of gender on navigation strategies revealed that females predominantly used the Non-turner strategy while males used both the Turner and the Non-turner strategy with comparable probabilities. Other than expected, video gaming experience did not influence strategy use. Based on a strong quantitative basis with the sample size about an order of magnitude larger than in typical psychophysical studies these results demonstrate that most people reliably use one out of three possible navigation strategies (Turners, Non-turners, Switchers) for spatial updating and provides a sound estimate of how those strategies are distributed within the general population.

## Introduction

Spatial orientation is a fundamental and complex cognitive process associated with nearly every movement of the human body in the environment. While most people manage navigational tasks without even consciously thinking about it, the underlying neuronal and cognitive mechanisms are poorly understood. Spatial navigation relies on using and integrating sensory information from different modalities (Berthoz, 1999; Rossier et al., 2000). However, different modalities initially encode their information based on different reference frames, with each reference frame providing its own coordinate system (Soechting and Flanders, 1992). There are at least two distinct frames of reference, namely the egocentric and the allocentric reference frame (Kolb et al., 1983). While the egocentric coordinate system is located within the agent and is contingent upon his orientation in space, the allocentric coordinate system decribes relations between objects, independent of the observers orientation (Klatzky, 1998). A spatial representation is then defined by a particular instantiation of reference frame use dependent on the task or environment. Redish and Touretzky (1997) proposed a model for animal navigation that included four different spatial representations; two of them are based on an egocentric reference frame, the other two on an allocentric reference frame. Human navigation might be governed by an interaction of various representations that are based on different frames of reference. Although humans are in general capable of using both reference frames, in most situations a preference to use either an egocentric or an allocentric strategy can be observed.

Spatial strategies, i.e., the use of a specific reference frame or the combination of different reference frames to solve a spatial task are thus to a large extend influenced by individual reference frame proclivities (Gramann, 2013). To illustrate this, let us consider the following example: you are visiting an unknown city looking on a city map in order to find your way. However, the map is not aligned with your current heading. In this situation you have at least two options to align your (egocentric) physical heading with the (allocentric) orientation of the map. First, you could rotate the map until it is aligned with your physical heading. Alternatively, you could mentally rotate until your imagined heading matches the orientation of the map. Why do some people turn the map while others mentally rotate and which cognitive mechanisms can be inferred from such behavior? Turning the map reflects a translation of the coordinate system of the map to match the egocentric coordinate system of the navigator's physical structure. Mentally rotating the navigator's heading reflects an adaptation to the allocentric coordinate system of the map. In other words, while one group of people prefers to compute spatial actions aligned with and based on their actual physical heading, another group prefers to compute spatial actions aligned with and based on a world-centered reference frame.

A promising way of investigating the cognitive basis for such differences in human behavior are virtual reality (VR) environments in which participants are free to use different reference frames when confronted with spatial tasks. Importantly, VR setups allow for precise experimental control. However, in typical desktop VR experiments, participants are not able to actively move and therefore lack embodied (proprioceptive and vestibular) cues. The absence of idiothetic information in VR experiments is associated with pronounced differences in spatial orientation strategies reflecting individual proclivities to use allocentric or egocentric reference frames (Riecke, 2008; Gramann, 2013). In particular, Gramann and colleagues demonstrated striking differences in the participants' responses when adjusting homing vectors after passages through virtual tunnels (Gramann et al., 2005, 2006, 2010).

In the categorization phase of this so-called “tunnel paradigm” participants saw virtual passages through tunnels with one turn to either the left or right side. At the end of a passage participants were ask to choose one out of two possible homing arrows to indicate their initial starting position at the beginning of the passage. Notably, one arrow indicated the direction toward the starting position based on an egocentric reference frame, corresponding to a change in cognitive heading during the turn. The other arrow indicated the homing direction based on an allocentric reference frame, corresponding to an unchanged cognitive heading aligned with the actual physical heading of participants. About half of the subjects preferred the egocentric homing arrow, while the other half preferred the allocentric homing arrow. However, the sample population was quite small and it remained unclear why participants used different reference frames for their responses.

Such differences in homing responses can be explained by individual proclivities to use different reference frames. However, Riecke and colleagues argue that the Non-turner behavior might be explained by simple left-right mirrored responses (Riecke, 2008). These studies however use a restricted range of path layouts. To systematically address this question in a recent study, Riecke (2012) used a wide range of path layouts confirming that differences in homing responses are based on a failure to integrate visually presented turns rather than due to “left-right mirrored responses.” Furthermore, accumulation of errors during the path integration process might contribute to behavioral differences if the error was systematic. Errors in the representation of the traversed path might be a result of incorrect encoding of the path or, alternatively, errors might result from an incorrect computation of a homing response based on a correct spatial representation. Fujita et al. (1993) proposed the encoding error model that is based on configural updating of spatial information. This model assumes that people encode an internal representation of the pathway, rather than a homeward trajectory. As a consequence, homing accuracy is proposed to decrease and reaction times to increase with increasing complexity of the path. While this might be true the model does not account for the systematic individual differences reported in earlier studies including more complex path layouts (Gramann et al., 2005; Plank et al., 2010). Other researchers suggested continuous updating models (Wiener, 2006) in which navigators continuously calculate a homing vector. This is in line with findings of Gramann and collegues (2006); Gramann et al. (2010) demonstrating that participants with distinct reference frame proclivities reveal differential neural activity already during the turning segment of the tunnel task. However, both the continuous and the configural model have difficulties in explaining the brain dynamic patterns and the performance differences of Turners and Non-turners in previous studies and future studies have to systematically address how the Turner and Non-turner behavior is connected to path integration strategies. The present study was designed to first investigate whether the individual differences in previous studies can be replicated for a large population. If this is the case, future studies can address the possible relationships of reference frame proclivities and underlying spatial updating processes.

Here we aimed at investigating how differences in strategy use are distributed in the overall population and which factors contribute to individual reference frame proclivities in a path integration paradigm. To this end, we analyzed data from a large number of participants to test whether previous findings on individual reference frame proclivities can be observed in a wider population and how such proclivities are distributed. Recently, the two main spatial strategies were replicated using a VR star-field task including horizontal (yaw) and vertical (pitch) rotation changes (Gramann et al., 2012). Besides two groups of Turner participants using an egocentric reference frame and Non-turner participants using an allocentric reference frame, some participants systematically switched from an egocentric reference frame for yaw rotations to an allocentric reference frame for pitch rotations. In the current investigation we thus included yaw and pitch rotations to further investigate whether this switching behavior can be replicated in a larger population. Moreover, we wanted to examine how stable reference frame proclivities are for individual participants and which factors potentially influence reference frame proclivities in the general population. In particular, we were interested how gender, video gaming experience, and self-estimated navigation abilities vary across strategy groups.

Gender differences are commonly proposed in navigation research. Most researchers agree that men outperform women on typical paper-and-pencil tests, or virtual navigation tasks that are based on geometric information (Moffat et al., 1998; Newhouse et al., 2007). Lawton (1994, 1996) evaluated self-reports of men and women and concluded that female participants less often use an allocentric strategy. Miller and Santoni (1986) and Dabbs et al. (1998) showed that females base their navigation decisions more on landmarks or egocentric information. Furthermore, it has been suggested that females perform worse when using allocentric-based strategies (Astur et al., 1998; Sandstrom et al., 1998; Saucier et al., 2002; Rizk-Jackson et al., 2006). Overall, the general consensus is that males show better performance and use more often allocentric strategies than females during navigation (Wolley et al., 2009). However, recently van Gerven et al. (2012) reported that when females are free to choose, they use allocentric strategies at least as often as males do, although using an egocentric strategy yielded to better performance. In order to shed more light on the issue, we aimed at investigating whether spatial reference frame proclivities differ between men and women selected from a large population.

Besides gender differences, recent studies imply an influence of video gaming experience on spatial cognitive processing. Most experiments focused on a correlation of video gaming experience and performance concluding that high video gaming experience leads to higher performance in navigational tasks (Frey et al., 2007; Richardson et al., 2011). Moreover, Smith and Du'Mont (2009) and Richardson and Collaer (2011) demonstrated that self-estimated video gaming experience is correlated with performance in virtual navigation tasks. However, as there is no direct evidence that video gaming experience influences the use of distinct spatial reference frames, we included a gaming-related questionnaire to get individual estimates of video gaming experience that further could be used to correlate with individual navigation strategies.

Several studies have shown that self-estimates of spatial abilities predict navigation performance reasonably well (Hegarty et al., 2002; Gluck and Fitting, 2003). To allow for analyzing the influence of self-estimates of spatial abilities on navigation strategies, we included two additional questions regarding self-estimated navigation abilities; the first question referred to the ability to use cardinal directions, the second to general navigation skills. Moreover, we recorded decision certainty with respect to participants' performance hypothesizing that self-estimated spatial navigation skills and decision certainty are more pronounced when participants demonstrated a clear and stable strategy compared to people that randomly applied different strategies. Furthermore, we aimed at investigating whether self-estimated navigation abilities and decision certainty vary between participants preferring different reference frames for navigation. In general, the correlation of objectively measured responses and subjective assessments is a quite promising approach and offers several benefits. Namely, existing variations in the subjective experience of participants can be understood and possibly related to observed differences in navigation behavior. These insights might thereby also be helpful to develop new technologies for the diagnosis or treatment of patients with impaired spatial abilities.

## Methods

### Online study

The experiment was designed as an online study. The URL to the study was “www.navigationexperiments.com/TurningStudy.html.” The main reason for designing an online study was the requirement for a large number of participants that could be best achieved through the internet. We advertized the online study on several web portals (e.g., Facebook, university homepage, etc.). Furthermore, we used existing scientific networks and contacted colleagues located in the US, Europe, and Asia to help acquiring participants. Therefore, we translated the webpage into six different languages (English, French, Spanish, Portuguese, German, and Russian). All participants performed the experiment independently on their own with detailed instructions given during the procedure. The homepage itself was programmed in HTML, Java Script, and CSS. The only requirement was that all participants used the latest version of Adobe's Flash player.

### Path integration task

**Figure 1:**
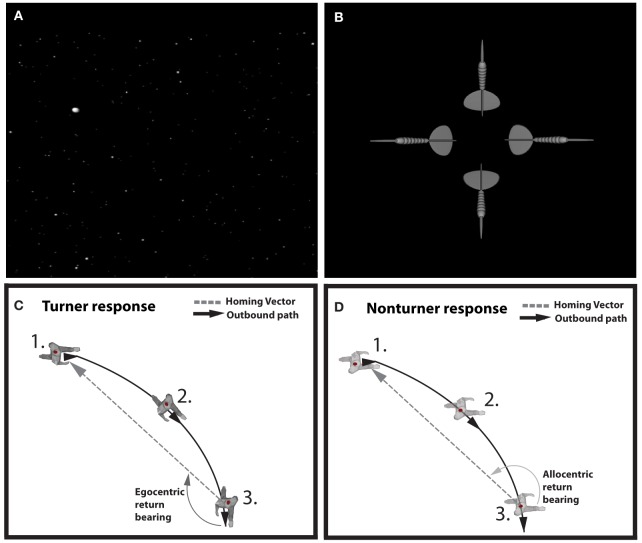
**Experimental paradigm. (A)** Snapshot of the star-field passage in the experiment. During the passage the white dots (stars)-induced visual flow indicating a turn into one direction. **(B)** Forced choice arrow selection in the experiment. After a 90° rightward turn. The arrow pointing to the right indicates the homing vector in line with an egocentric reference frame; the arrow pointing to the left is congruent with an underlying allocentric reference frame. Choosing one of the other two arrows (up and down in this case) was counted as incorrect response. **(C,D)** Turner and Non-turner responses respectively in a schematic drawing of a 90° rightward turn from a bird's eye view. The curved black line indicates the turn of the virtual passage. (1) Before the turn both strategy groups Turners (panel **C**) and Non-turners (panel **D**) have an identical heading. (2) While the cognitive heading of Non-turners does not change during the stimulus turn, Turners adapt their cognitive heading according to the degree of the stimulus turn. (3) At the end of the passage after the turn, Turners base their response on the cognitive heading aligned with the virtual environment and therefore point to their right and back (egocentric bearing return). Non-turners respond based on a cognitive heading that is still aligned with their physical heading and thus point to their left and back (Allocentric return bearing).

The main purpose of the study was to investigate strategy differences in virtual path integration. Following the idea of the tunnel paradigm, participants saw videos of passages through a dot cloud (Figure 1A). Every passage consisted of a first straight segment followed by one rotation (stimulus turn) to the left/right for yaw trials or up/down for pitch trials. After the stimulus turn a second straight segment followed after which the passage ended. The videos were created using “Vizard 3.0®,” converted into .mp4 format and displayed with “Flowplayer 3.2®.” The different segments smoothly transitioned and one passage including all three segments (first straight segment, stimulus turn, last straight segment) was perceived as continuous visual flow. Altogether three different angels (30, 60, and 90°) and four different directions (up, down, left, and right) were realized, adding up to 12 different passages. Each passage was presented twice such that all participants watched 24 videos in a randomized order. At the end of a passage four homing arrows appeared pointing into different directions (Figure 1B). Two out of the four displayed homing arrows were considered correct. Both indicated the exact direction in three dimensional space toward the starting location dependent on the path traversed. The orientations of the displayed homing arrows dependent on the angle of rotation during the passage such that passages with acute angled turns resulted in homing arrows pointing more inward than passages with less acute turning angles. However, one arrow pointed back in accordance to an allocentric reference frame while the other was in accordance to an egocentric reference frame. Choosing either of the other two arrows was considered as an erroneous response. For example, after a passage with a rotation in the yaw plane the two homing arrows pointing back-left or to back-right were considered correct (Figures 1C,D), while the two homing arrows pointing back-up or pointing back-down were considered incorrect (and vice versa for pitch rotations). Participants were then asked to select one out of the four homing arrows to indicate the direction toward the starting position. This was done by clicking on the respective arrow with a computer mouse. We measured response type (egocentric, allocentric, or incorrect) and reaction time. Reaction time was defined as the time from onset of the arrow selection screen until the participant clicked on one of the arrows. This was done with a Java Script file running on the client PC, which ensured that there was no bias from internet connection. Inter-trial time was not recorded as it was heavily biased by the speed of the individual internet connection. Subjects were instructed to start the experiment only when sufficient time was at hand to perform it in a single session. In that case the experiment took about 15–20 min in total. Before the experiment started all subjects were informed about the task and instructed to focus during the whole experiment and not take any breaks (see appendix for the instructions). After participants navigated through all passages they filled out a questionnaire asking for gender, age, gaming and computer experience, decision certainty, and self-estimated navigation skills (see Appendix for the complete questionnaire).

### Participants

Data from 300 participants was recorded. Six subjects were excluded as they had missing data in the questionnaire. Initially, we analyzed the distribution of incorrect responses. Most subjects did not commit any error but some subjects responded incorrectly in more than half of the trials indicating complete spatial disorientation or a lack of attention during the task. Because we wanted to investigate homing adjustments in participants that were oriented during the task, we excluded subjects with too many incorrect responses. The distribution of incorrect responses showed a natural inflection point at 2–3 incorrect responses and we thus removed subjects with more than 2 incorrect responses (*n* = 34) from further analyses. This resulted in 260 participants with nearly an even split of male (132) and female (128) participants. Most participants were university students with an average age of 27.14 years (SD = 9.83 years) living in more than 15 different countries. Cultural impact on spatial navigation was not analyzed due to the relatively sparse distribution (most participants lived in Germany or Spain). In total, 24 of all 260 participants were left handed. No participant received reimbursement for the experiment but all participants were offered information on their preferred spatial strategy at the end of the experiment.

### Correlation of behavioral and questionnaire data

We preprocessed questionnaire data dependent on data scaling; dichotomous variables were dummy coded and variables that used a *Likert Scale* were normalized and then z-transformed. (See appendix for the complete questionnaire). Overall we investigate the correlation of preferred spatial strategy with the factors: gender, video gaming experience, sense of direction, general navigation self-estimation, and decision certainty. As the study was conducted online, we did not include a complete spatial navigation questionnaire (e.g., Santa Barbara Sense of Direction Scale) because it would have taken much time and some subjects presumably would have left the page without filling out the complete questionnaire. Hence, we included only 2 questions. One question was targeting allocentric (cardinal direction) navigation performance and another question was targeting general navigation performance (see appendix for whole questionnaire).

For video gaming experience we combined the responses of five questions into one final estimate for each subject. Since the different sub-scales included different scaling, all variables were first normalized and then a PCA was computed to reduce dimensionality. The z-transformed value of the first principle component was finally used for the analysis of video gaming experience.

#### Discriminant analysis

Strategy was analyzed by discriminant analysis as a function of the following factors: gender, self-estimated video gaming experience, self-estimated ability to use cardinal directions, self-estimated general navigation skill, and decision certainty. Other factors like age or handedness could not be included due to sparseness or rareness. In order to identify which combination of individual factors distinguished the strategy groups best, we calculated the structure matrix of the discriminant functions. Because the dependent variable (strategy use) had four levels (Turner, Non-turner, Switcher, No Preference) the discriminant analysis computed 3 different discriminant functions, providing a weighted combination of the independent variables that led to the maximal separation of the four levels of the dependent variable. Finally, we tested each discriminant function for significance.

#### Analysis of variance

The discriminant analysis provided us with a linear combination of individual factors that discriminated between strategy groups. Wilks' Lambda was computed to test for significance of the individual variables. Additionally, we inverted the analysis using gender, gaming experience etc., as the dependent variable and strategy group as the independent variable. With that approach we were able to apply an analysis of variance with pairwise comparisons in order to investigate the exact difference of these factors between the strategy groups.

#### Classification

The final step in the analysis was to predict strategy based on the questionnaire data. First, we used the discriminant functions for classification. Such generative models are well-suited since they describe the relationship between the predictor variables and different groups of the dependent variable. A second approach was to use a Support Vector Machine (SVM) instead of discriminant functions. The advantage is that a SVM can also calculate non-linear relationships and use those weights for later classification. In both methods we used cross validation to estimate prediction accuracy.

## Results

### Response behavior

**Figure 2:**
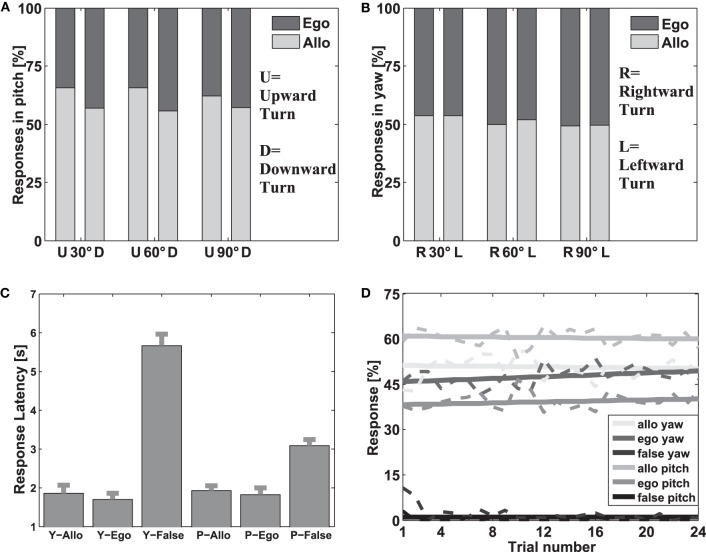
**Investigation of response types. (A,B)** Ratio of allocentric vs. egocentric responses for all passages in pitch (panel **A**) and yaw (panel **B**). In both figures the y-axes displays the relative amount of allocentric vs. egocentric responses. The x-axes indicate the different passages in both yaw and pitch. The dark gray bars indicate the amount of egocentric responses; the light gray bars indicate the amount of allocentric responses. **(C)** Response times for all responses types. The y-axis shows the response times in seconds from stimulus onset (appearance of the arrows) until one arrow was chosen (mouse click). The x-axis shows the various response types (allocentric, egocentric, and incorrect) separately for both axes (Y stands for yaw and P for pitch; False stands for incorrect). The gray bars indicate the means and standard errors of response times for each response type. **(D)** Distribution of response types over trials. The y-axis displays the relative amount for all response types. The x-axis shows the trial number. Each line represents the relative amount of the respective response type for each trial in yaw or pitch. The dotted lines display the actual data and the solid lines show the linear regression fits.

First we analyzed the distribution of response types over the course of the experiment, investigating whether strategy-specific responses occurred more often in the beginning of the experiment as compared to later trials or vice versa. The relative amount of each response type over trials was then fitted with a linear regression and the slopes of the regression lines were tested in an F-test against the zero hypotheses of zero slope. We observed a stable distribution of allocentric, egocentric, and incorrect responses for both axes over the course of the experiment (Figure 2D). All slopes of the linear regression curves did not significantly differ from zero (*p* > 0.05) for any response type.

Due to the nature of the online experiment we took additional measures to ensure that only trials entered further analysis where participants likely attended to the task and successfully kept up spatial orientation. First, a Two Way ANOVA with factors axis (yaw and pitch) and response type (egocentric, allocentric, and incorrect) was computed to uncover potential differences in response latencies. The analysis revealed a significant influence of the factor response type [*F*_(1, 765)_ = 12.98; *p* < 0.01] but not for the factor axis [*F*_(1, 765)_ = 0.68; *p* > 0.1] or an interaction of both factors [*F*_(1, 765)_ = 2.17; *p* > 0.1; Figure 2C]. An additional pairwise comparison demonstrated that incorrect responses had significantly higher response latencies than both egocentric and allocentric responses [*F*_(2, 765)_ = 40.39; *p* < 0.01]. As this shows that incorrect responses were somehow different from correct responses, we did not analyze incorrect trails for the rest of the analysis.

As incorrect responses were not considered further, the amount of allocentric responses was the inverse of the amount of egocentric responses. In a next step we analyzed the ratio of allocentric and egocentric responses for different passages. In order to be able to perform statistical analysis, we separated the data into 10 groups of 26 participants each. For each of these groups we then calculated the mean (egocentric vs. allocentric) response ratio for different passages. An ANOVA with factors angle (30, 60, and 90°), axis (yaw, pitch) and order (first or second presentation) investigated the difference in response type (allocentric vs. egocentric). The analysis revealed a significant main effect for the factor axis [*F*_(1, 108)_ = 74.2, *p* < 0.001] but no effect for the factor angle [*F*_(2, 108)_ = 2.55, *p* = 0.0831] and the factor order [*F*_(1, 108)_ = 1.56, *p* > 0.1]. Furthermore, none of the interactions reached significance. Comparing Figures 2A (pitch) and B (yaw) demonstrates that participants used the allocentric strategy more often in pitch than in yaw passages. This result underlines that reference frame proclivities are dependent on the environment, i.e., the axis of rotation. On that account, passages with different angles and directions were aggregated and only the factor axis (yaw vs. pitch) was considered for further analyses. This way the number of different response types was reduced to four (egocentric yaw, allocentric yaw, egocentric pitch, allocentric pitch).

### Reference frame proclivities

**Figure 3:**
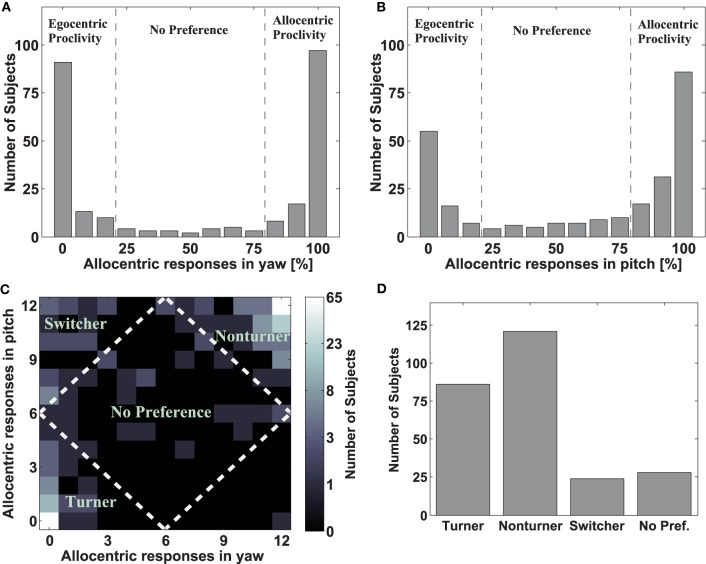
**Response distribution and strategy formation. (A,B)** Histograms of responses for yaw (panel **A**) and pitch (panel **B**) rotations. In both figures the y-axes indicate the number of subjects. The x-axes show the amount of allocentric responses. The height of the gray bars indicates the amount of subjects for each ratio of allocentric vs. egocentric responses (0 means all responses were given using and egocentric reference frame; 100 means all responses were given using an allocentric reference frame). The black dashed lines indicate the boundaries that were later on used to group people into different strategy classes. **(C)** Combined response pattern for all subjects indicating strategy class. The y-axis indicates the amount of allocentric responses in pitch, the x-axes indicates the amount of allocentric responses in yaw. The brightness of the squares reflects the amount of subjects that have the identical ratio of allocentric vs. egocentric responses for both yaw and pitch (bright colors indicate many subjects; dark colors few subjects). The logarithmic scale to the right shows the amount of subjects corresponding to each level of luminance, with rounded values. The white dashed line marks the boundaries between the strategy groups. Additionally, the labels indicate the names for the strategy groups. **(D)** Strategy distribution in the overall population. The y-axis indicates the amount of subjects; the x-axis indicates the different strategy groups. The height of the gray bars indicates the amount of subjects belonging to a particular strategy group.

After we performed all the necessary preprocessing steps we investigated how stable reference frame proclivities were on an individual basis. As the histogram in Figure 3A shows, most participants exclusively chose either an allocentric homing arrow (rightmost bar) or exclusively an egocentric homing arrow (leftmost bar) in yaw trials. We observed a similar pattern for pitch trials (Figure 3B). As each passage was displayed twice, we compared reference frame proclivity for the first and second response of each passage. Hence the Pearson product-moment correlation coefficient *r* was calculated for both yaw (*r* = 0.9563) and pitch (*r* = 0.9166) trials. The correlations were significant for both axes (*p* < 0.001). Moreover we also calculated the correlation between yaw and pitch responses with respect to reference frame proclivity. Again the Pearson product-moment correlation coefficient (*r* = 0.81) revealed significance (*p* < 0.001). These data indicate that participants responded consistently with clear preferences throughout and that reference frame proclivity did not change over time.

### Strategy formation

The key interest in the present study was to evaluate the distribution of navigation strategies in the overall population. In order to make a more general statement about individual strategy use, the data was synthesized with respect to the overall response pattern in yaw and pitch axes simultaneously. Most participants demonstrated a clear and stable navigation strategy, as reflected in two clusters in the lower left and the upper right corner (Figure 3C). Only few participants were located in the center of the distribution, showing no preference in reference frame use. Instead the peaks of the clusters were located on the edges of the distribution, reflecting the fact that most participants exclusively chose either an egocentric or an allocentric reference frame for both yaw and pitch rotations.

We classified all subjects into one of five strategy groups using the factors reference frame proclivity (egocentric vs. allocentric) and axis (yaw vs. pitch). Participants who used an egocentric reference frame in at least 19 out of trial 24 trials (>75%) were classified as Turners, while participants using an allocentric reference frame in at least 19 out of 24 trials (>75%) were classified as Non-turners (for a detailed discussion on the nomenclature see Gramann et al., 2012). In addition, participants could switch between reference frames dependent on the axis of rotation. In theory two scenarios were possible: Participants could switch from an egocentric reference frame in yaw to an allocentric reference frame in pitch or, vice versa, participants could switch from an allocentric reference frame in yaw to an egocentric reference frame in pitch. Participants adopting a yaw-ego pitch-allo strategy in at least 19 out of 24 trials were classified as Switchers, while participants adopting a yaw-allo pitch-ego strategy were classified as Inverse Switchers. However, as only one subject adopted the latter strategy we did not further investigate this behavior. The remaining participants revealing no clear reference frame proclivity were assigned to the No Preference group. This classification scheme conforms to the use in previous experiments (Gramann et al., 2010, 2012).

Finally, we calculated the number of participants in each strategy group. As shown in Figure 3D, 33.1% (86 subjects) were classified as Turners (egocentric reference frame proclivity for both axes), 46.5% (121 subjects) as Non-turners (allocentric reference frame proclivity for both axes). Furthermore 9.2% (24 subjects) consistently switched between an egocentric reference frame in yaw and an allocentric reference frame in pitch. However, the reverse assignment was rarely observed and only a single participant switched consistently between an allocentric reference frame in yaw and an egocentric reference frame in pitch. Hence this subject is not considered in further analysis while participants consistently switching from egocentric in yaw to allocentric in pitch were now labeled *Switcher*. In total, 10.8% (28 subjects) did not show a strategy preference. These data show that the overwhelming part of subjects responded consistently using either the Turner, Switcher, or Non-turner strategy.

### Response latencies

**Figure 4:**
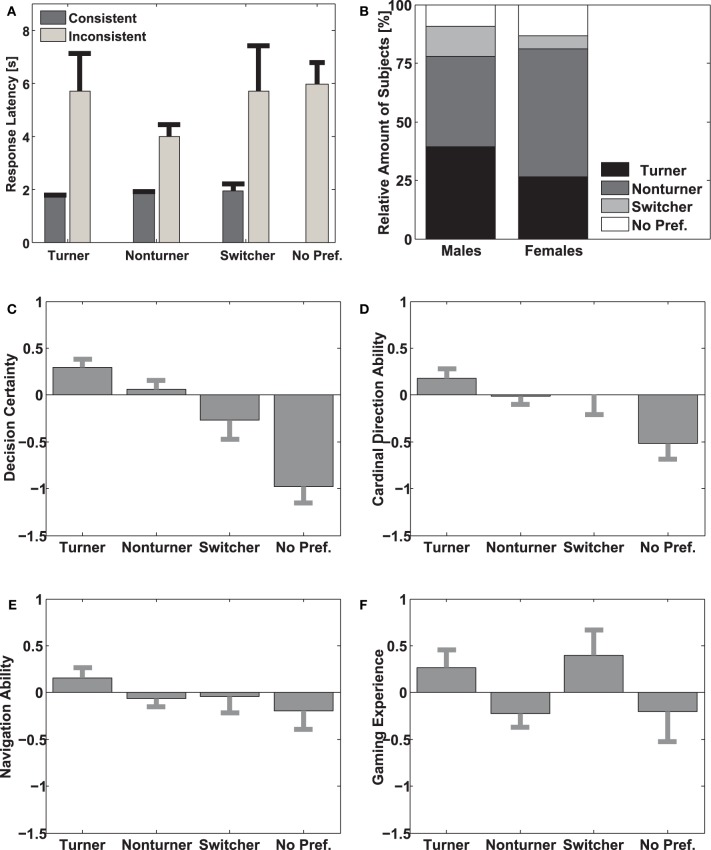
**Subgroup investigations (A) Response latency for strategy groups.** The y-axis indicates the response time in seconds. The x-axis represents the four different strategy groups. The dark gray bars show the average response latency for responses inconsistent with the preferred strategy including standard errors; the light gray error bars show the average response latency for responses consistent with the preferred strategy; also including standard errors. **(B)** Distribution of strategy use for males and females. The y-axis indicates the relative amount of subjects belonging to a particular strategy group. The x-axis separates male and female participants. The stacked bar diagram shows the distribution of strategy groups for male and female subjects. The different shades of gray color-code the four different strategy groups. **(C,E)** Variations of self-assessed variables between strategy groups. The y-axes indicate the value of the self-estimated variable on a z-score scale. The x-axis separates the different strategy groups. The height of the gray bars indicates the mean value for the different strategy groups, including standard errors. Panel **(C)** displays *Decision Certainty;* panel **(D)** displays *Cardinal Direction;* panel **(E)** displays *Navigation Skill;* and panel **(F)** displays *Gaming Experience*.

In a next step we analyzed response latency as a function strategy and consistency. In particular, we wanted to know first whether different strategy groups answered faster or slower than others and second whether trials in line with the overall preference of a subject have a shorter reaction time than trials that were not. To this end, we separated all participants into 10 groups with 26 participants each. For each of these groups we divided the data according to the two factors of interest, i.e., strategy use and consistency. As for subjects in the no-preference group a separation of consistent and inconsistent trials is not possible, we visualized results of these subjects but excluded them from the statistical analysis. Next we calculated the median response time pooled over subjects within each group but separately for each combination of conditions. This resulted in 10 (groups) × 3 (strategy use) × 2 (consistency) values. As the median is a robust estimator of central tendency, this procedure ensured that outliers did not influence the results. Figure 4A visualizes the mean of the 10 groups, separately for each condition also including the no-preference group. The figure shows pronounced differences between consistent and inconsistent responses for all strategy groups. Moreover, average reaction times in the no preference group are more similar to inconsistent than to consistent responses of the other strategy groups. Finally, we performed a Two Way ANOVA with the factors strategy and consistency as independent variables and reaction time as the dependent variable. In order to avoid a bias from the initial splitting of subjects into the 10 different groups and to provide more robust statistics, we iteratively tested 10,000 different splittings of subjects and gathered the distribution of *p*-values from the subsequent ANOVAs. For the factor consistency all *p*-values were below 0.05 and 97.89% were below 0.01 showing that consistent responses were significantly faster than inconsistent responses. No other factor or interaction reached significance (in both cases 98% of *p*-values failed to reach *p* < 0.05). Representative One-Way ANOVAs revealed a significant effect of consistency for all strategy groups [Turners: *F*_(1, 19)_ = 7.85, *p* < 0.05, Non-turners: *F*_(1, 19)_ = 23.91, *p* < 0.01, and Switchers: *F*_(1, 19)_ = 4.74; *p* < 0.05]. These results support the assumption that Switchers indeed constitute a strategy group separate from Turners and Non-turners. Furthermore, these results further support the hypothesis that the use of a non-preferred spatial reference frame is associated with different cognitive processes that are likely computationally more demanding.

### Strategy and self-assessment

Variations in navigation behavior were most prominent between but not within subjects. To uncover this disparity we performed a discriminant analysis and consequently calculated the structure matrix as shown in Table 1. The analysis revealed that the first discriminant function significantly differentiated between the strategy groups (df = 12, Wilks' Lambda = 0.816, *p* < 0.001), while the second discriminant function was borderline significant (df = 6, Wilks' Lambda = 0.954, *p* = 0.062). The third discriminant function did not help differentiating strategy groups (df = 2, Wilks' Lambda = 0.999, *p* > 0.1). The first function was mostly dependent on *decision certainty* and *cardinal direction proficiency*, while the second function was mostly dependent on *gender* and *video gaming experience*. Furthermore, we investigated significance of the single variables using Wilk's Lambda in an ANOVA *F*-test. The result showed that *gender, cardinal direction proficiency*, and *decision certainty* significantly contributed to the discrimination of the strategy groups (*p* < 0.05). However, to gain more insight about the relation of the predictor variables and the strategy groups we analyzed whether the values of the predictor variables were significantly different between the strategy groups. The analysis of *gender* (Figure 4B) revealed that females were predominantly Non-turners (54.7%) as compared to Turners (26.6%), while males used both the Turner (39.4%) and the Non-turner (38.6%) strategy more or less evenly distributed. Furthermore, males were more likely to be Switchers (12.9%) than females (5.5%). Overall, Turners had the highest *decision certainty* followed by Non-turners, Switchers, and finally participants with no reference frame preference (Figure 4C). A pairwise-comparison revealed a significant difference between Turners and Non-turners compared to subjects with no strategy preference [*F*_(3, 258)_ = 14.05, *p* < 0.001]. The *self-estimated ability to use cardinal directions* revealed a similar pattern as *decision certainty*. Turners revealed the highest confidence in their ability of cardinal direction use and participants without strategy preference were most unconfident using cardinal directions. Again a paired comparison revealed significant differences between Turners and Non-turners compared to subjects without preference. [*F*_(3, 258)_ = 3.49, *p* < 0.05, Figure 4D]. Surprisingly, self-estimated *general navigation skills* did not show any difference between strategy groups [*F*_(3, 258)_ = 1.19, *p* > 0. 1; Figure 4E]. Although there is a trend that video gaming experience differentially affected strategy use for males and females the ANOVA revealed that *video gaming experience* neither directly [*F*_(3, 258)_ = 2.05, *p* > 0. 1] nor via an interaction with gender-influenced strategy use [*F*_(19, 259)_ = 0.83, *p* > 0.1; Figure 4F].

**Table 1:** **Structure Matrix of Discriminant Functions**.

**Predictor variable/discriminant function**	Function 1^*^	**Function 2**	**Function 3**
Decision certainty	**0.970**	−0.002	0.150
Cardinal direction ability	**0.466**	−0.242	−0.146
Gender	−0.183	**0.951**	0.170
Gaming experience	0.118	**−0.668**	0.307
Navigation skill	0.222	−0.261	**0.784**

The first column indicates the name of the predictor variable. The second to forth column show the structure coefficients for the three different discriminant functions. The values in bold depict the highest correlation of a certain predictor variable with a certain discriminant function. The asterisk indicates significance of the discriminant function.

### Strategy prediction

Applying results of the previous section we investigated in how far the chosen strategy can be predicted based on the self-assessment. Predicting individual reference frame proclivity separately for both axes using a SVM reached 62.38% in yaw and 67.54% in pitch, compared to a chance level of 50%. Predicting complete strategy groups in 3D space (in Turner, Switcher, Non-turner, and inconsistent groups) using discriminant functions, classification performance was above chance (25%) at a level of 42.1% correct classifications. SVM classification improved this result to a correct classification rate of 54.36%. These data demonstrate that the chosen strategy can be predicted based on the self-assessment at a moderate level.

## Discussion

Here we investigated a large group of participants in a 3D virtual navigation task and found strong evidence that the majority of subjects reliably used a particular navigation strategy. More than 85% of the sample population demonstrated a clear and stable strategy in the experiment. Although most subjects preferred the same reference frame for both axes, we proofed the existence of a third distinct strategy group that systematically switched between reference frames from egocentric in yaw to allocentric in pitch. The opposite change of reference frame proclivities was rarely observed and did not represent an own strategy group. We conclude that there are three distinct spatial strategies (Turners, Non-turners, and Switchers) and that subjects reliably choose one spatial strategy for virtual navigation. These results support earlier findings concerning the use of distinct spatial strategies (Moffat et al., 2001; Gramann et al., 2005, 2006, 2010; Riecke, 2008; Rodgers et al., 2012; Gramann, 2013). Most importantly, we showed that the reported difference in strategy use is a general and remarkably prominent phenomenom in the overal population. To our understanding such strong distinctions during updating of spatial information in yaw and pitch navigation must be considered in future studies of spatial navigation.

### Potential restrictions of the present study

The experiment was conducted as an online study that is potentially associated with a number of issues. First, participants were not directly instructed and supervised by an experimenter. This opens the possibility that participants might not have understood the task or could have been distracted (people walking in the room, phone calls, etc.) during the experiment. However, instructions were given in detail on the webpage beforehand. Furthermore, it is reasonable to assume that participants who did not understand the task or were not concentrated during the experiment would have made many incorrect responses. However, most subjects had fast reaction times and did make few if any errors. Specifically, the few subjects with more than two errors were excluded from the analysis. Furthermore, the experiment was self-scheduled by the participants, favoring a timing-free of other constraints or tasks. Therefore, we can reasonably assume that all participants used in the analysis attentively carried out the task as would subjects under lab conditions.

Second, an online study does not control for the experimental environment during the task. Participants in this study presumably used different computers, monitors, and software (browser). However, we tested various browsers and monitors before starting the experiment and did not observe any crucial differences. Furthermore the position between the monitor and the participant might have varied between participants. However, we do not have any reason to assume that it conflicts with the classification schema (Turner, Non-turner, and Switcher) made in our paradigm. Furthermore, it is reasonable to assume that if the angle between monitor and participants varied that such a bias is normally distributed among the population (i.e., some people place it to their right, most in the center, some others to their left). Finally, there is no evidence suggesting that certain subgroups (e.g., males, females) have a preference for a particular option (monitor position). Overall it is to say that the task was relatively simple and we did not get any critical feedback about technical problems. Altogether we believe that the benefits of the online study outperformed the shortcomings by far.

### Individual differences influencing strategy selection

A major result of the present study is a gender-specific difference in spatial strategies. Most former studies mentioned that males prefer allocentric navigation while women tend to use egocentric strategies (Lawton, 1994, 1996). However, van Gerven et al. (2012) recently proposed that women use allocentric strategies to a similar extent than males. This is supported and extended by our results demonstrating that the majority of women in our task prefer an allocentric navigation strategy and that more women than men prefer an allocentric over an egocentric navigation strategy. One might specultate why we found such differences while earlier studies did not? One reasons might be that previous studies forced participants to use one or the other spatial strategy. Based on such instructions Sandstrom and colleagues come to the conclusion that women avoid allocentric strategies because their performance was impaired compared to egocentric strategies (Astur et al., 1998; Sandstrom et al., 1998; Saucier et al., 2002; Rizk-Jackson et al., 2006). This conclusion is at odds with the observed distribution of spatial strategies in a large population of participants. Furthermore, in our study, video gaming experience varied significantly between men and women; however it did not have a significant influence on strategy use. Therefore, the reported gender differences cannot be explained by different levels of experience with virtual environments.

Richardson et al. (2011) and Frey et al. (2007) demonstrated that video gaming experience is highly correlated with performance in virtual navigation tasks. Here we investigated the influence of video gaming experience on the use of distinct spatial strategies. More specifically, we expected participants with a strong gaming background to be more easily immersed within the virtual environment and thereby to adopt an egocentric strategy. However, no such effect was observed. One explanation for the missing influence of video gaming experience on spatial strategies could be the experience with different types of computer games and the associated reference frames (e.g., 3D-first person vs. 2D-bird's eye view games). Conclusively, some video games favor an egocentric strategy while others favor an allocentric strategy. Hence, high video game experience could lead to different biases in strategy use depending on the type of game that individuals prefer. In order to determine the influence of video gaming experience on navigation strategies, future studies have to use extended questionnaires analyzing differences in reference frames used in specific video games.

Finally, we observed significant variations between strategy groups for decision certainty and cardinal direction abilities. The fact that people with no reference frame proclivity were much less confident in their responses compared to Turners and Non-turners suggests that those subjects indeed did not follow a clear navigation strategy and therefore supports our categorization of strategy classes. Gluck and Fitting (2003) and Hegarty et al. (2002) reported that individual estimates of navigation abilities correlate with the observed performance in spatial tasks. Here we speculated that different levels of spatial performance correlate with the use of distinct reference frames. Although cardinal direction use implies an allocentric reference frame, we did not find differences between Turners and Non-turners but again between participants revealing a clear and stable navigation strategy as compared to participants who did not show any preference. In general, these results suggest that both Turners and Non-turners are able to use (allocentric) cardinal directions in the real world, while people without strategy preference are in general more challenged during spatial orienting.

Overall the influence of individual differences in established spatial measures on reference frame proclivities did not match all our hypotheses. In particular, it remains unclear which other important factors contribute to the general distinction between Turners and Non-turners. However, we have shown that a weighted linear combination of the variables gathered in this study is able to significantly discriminate between strategy groups and helps to predict strategy use up to a reasonable level. How such a reference frame proclivity observed in a VR setup is related to real world navigation remains speculative. In a current experiment we are investigating whether the strategy differences observable in virtual path integration also apply to real world navigation. In particular we are interested whether Non-turner responses are also present during real world navigation. However, the influence of reference frame proclivities might also be reflected in other ways. Potentially Turners tend to use well-known routes instead of computing detours in cases where this might be possible or Non-turners might prefer to communicate directions based on allocentric information (e.g., cardinal directions). Future research has to investigate these issues.

### Reference frame proclivities in yaw and pitch

We provide evidence that reference frame proclivities in a virtual path integration paradigm are dependent on the specifics of the environment, i.e., the axis of rotation. However, the question remains, why some participants use such a Switcher strategy. Vidal et al. (2004) showed that performance in a path recognition paradigm was better in terrestrial navigation including only yaw rotations, compared to weightless navigation also including pitch rotations. Human navigation is innately specialized for terrestrial (horizontal) navigation and performance in pitch trials could be impaired because of two main factors (Gramann et al., 2012). First, yaw rotations have a higher ecological validity (we experience them more often in the real world) and second the conflict between vestibular and visual information is more pronounced in pitch as compared to yaw rotations. While for yaw trials there is (only) a mismatch in the rotation information in pitch trials there is an additional mismatch in with respect to gravitational forces (Gramann et al., 2012). Using a similar paradigm Gramann et al. (2012) found that both absolute pointing errors and pointing variability were only slightly increased in pitch trials as compared to yaw trials. However, in the current experiment participants used significantly more often the allocentric strategy in pitch compared to yaw trials. Arguably, a decrease in pitch performance, arising from an increased visuo-vestibular conflict, might be counterbalanced or avoided by a shift of reference frame use. Strong evidence for this assumption comes from participants preferring an egocentric reference frame in yaw but switched to an allocentric reference frame in pitch, but not vice versa. Divers, pilots, and certain athletes who are used to vertical head rotations provide a good opportunity in this respect for future research.

## Summary

Altogether the present study provides strong evidence that humans have clear and stable reference frame proclivities for updating spatial information on a single axis. Differences in reference frame proclivities between both axes (yaw and pitch) are potentially related to the difference in the ecological validity of both kinds of rotations (horizontal vs. vertical). Individual responses based on the non-preferred reference frame with respect to participants' overall preference demonstrated prolonged response latencies, indicating differences and/or higher effort with respect to the underlying cognitive processes. Combining both yaw and pitch reference frame proclivities makes up three distinct and separable spatial strategies (Turners, Non-turners, and Switchers). More than 85% of participants reliably chose one of these strategies to solve the 3D path integration task. The fact that our study comprises data from 300 subjects renders it likely that the reported distribution of navigation strategies is a robust estimate of the true variation within the overall population. Contrary to earlier studies, we find that women prefer the Non-turner strategy that is based on an allocentric reference frame, while men do not show a preference between the Turner and the Non-turner strategy. Furthermore, we demonstrate that a linear combination of the variables gender, decision certainty, and cardinal direction proficiency can be used to discriminate strategy groups and also predict group membership to some degree. In future research, we aim to further investigate the influence of other factors, i.e., age and cultural background on reference frame proclivity to finally unravel the underlying factors determining human navigation strategies.

### Conflict of interest statement

The authors declare that the research was conducted in the absence of any commercial or financial relationships that could be construed as a potential conflict of interest.

## Appendix

### Experimental procedure

1. **Start the video**: In this experiment you will see 24 short videos of virtual passages through a star-field. After you start the experiment the first video will be loaded. When it's loaded you can start it by clicking on the arrow in the middle of the screen. The video will now start in full-screen mode. Please move your mouse cursor to the lower edge of the screen so that it disappears.
2. **What you will see**: All videos show passages through star-fields and each video will start with a black screen. Then a countdown will indicate the start of the passage. Every passage through a star-field starts with a straight segment and ends with a straight segment. In the middle of a passage, after the initial straight segment, there will be a turn. A turn can be to the left or to the right, or it can go up or down. Your task is to keep up orientation and to indicate, at the end of the passage, where the starting position of the passage was.
3. **How you respond**: At the end of a passage four arrows will appear pointing into different directions. Please select the one arrow that correctly points back to your starting position. Click on the arrow that you intuitively think is correct. When you lost your orientation during the passage please choose the arrow that you think is the most likely one. When you are done with the arrow selection, the next trial starts by loading another video.
4. **Questionnaire**: After the last trial there will be a short questionnaire on your experience with the experiment. Please fill out the required passages and finally press the submit button at the end of the page.

Please note that during the experiment you should *never use the “backward button” in your browser*, since it will bring you back to the start page and all data will be lost!

### Final questionnaire

Please fill out the questionnaire below. It will take you approximately 5 minutes and it's mandatory for further participation.

Age

Gender

✯ male
✯ female

Handedness

✯ right
✯ left

Occupation

Nationality

In which country did you grow up?

How much time on average do you spend on a computer per day?

➢ Less than 30 minutes
➢ less than 1 hour
➢ between 1 and 2 hours
➢ between 2 and 3 hours
➢ between 3 and 5 hours
➢ between 5 and 8 hours
➢ more than 8 hours

Have you ever played video games?

✯ Yes
✯ No

Do you currently play video games?

✯ Yes
✯ No

How long have you been playing video games?

➢ <6 month
➢ 1 year
➢ 2–5 years
➢ 5–10 years
➢ 10 years or more

How often (approximately) do you currently play video games?

➢ Daily
➢ weekly
➢ once a month
➢ once in 6 month
➢ once a year
➢ less than once a year or never

How good do you feel you are at playing video games?

➢ Very good
➢ moderately good
➢ not very skilled
➢ no skill

How confident were you about your answers?

Very unconfident (1) - very confident (7)

Were there any problems during the experiment?

Many problems (1) - no problems (7)

Did you like the experiment?

No, I did not like it at all (1) - Yes, I liked it a lot (7)

How do you estimate your own spatial navigation skills?

Very bad (1) - very good (7)

How confident are you using cardinal directions?

Very unconfident (1) - very confident (7)

Would you like to participate in a follow up study?

✯ Yes
✯ No

Is it your first time participating in this study?

✯ Yes
✯ No

Do you have any further comments, problems or suggestions?
